# Visuospatial processing: A review from basic to current concepts

**DOI:** 10.1590/S1980-57642014DN82000014

**Published:** 2014

**Authors:** Eduardo Sturzeneker Trés, Sonia Maria Dozzi Brucki

**Affiliations:** 1 MD, Neurologist, Resident of Cognitive and Behavioral Neurology, University of São Paulo, SP, Brazil; 2 PhD, Neurologist, Hospital Santa Marcelina; Cognitive and Behavioral Neurology Unit, University of São Paulo, SP, Brazil

**Keywords:** visuospatial functions, processing, pathway, function

## Abstract

**Introduction:**

Visuospatial processing is a fundamental aspect in human cognition, belonging to a complex and intricate network. It is, in other words, one of the building blocks of an individual's identity and behavior.

**Objective:**

To allow an overall and updated review of visuospatial processing and its related events, in light of new techniques and evidence, focusing on basic concepts of higher cortical functions, its pathways and associated systems.

**Methods:**

The study was conducted based on the national and international databases LILACS, MEDLINE, ScieLo and Pubmed; using the search word "visuospatial" in combination with "pathway", "processing", "function", "fMRI" and "attention".

**Results:**

A total of 77 references deemed relevant for its historical, conceptual or updated relevance were selected out of 1222 retrieved; including English, Spanish and Portuguese languages. A critical review was carried out and many new aspects discussed.

**Conclusion:**

A new functioning and construction of sight processing is being shaped, culminating now in a model based on dynamic and integrated interactions between pathways and systems.

## Abstract (pt)

**Introdução:**

O processamento visuoespacial é um aspecto fundamental da cognição humana, pertencendo a uma complexa e intricada rede. É, em outras palavras, uma das pedras fundamentais da identidade e comportamento de um indivíduo.

**Objetivo:**

Permitir uma revisão geral e atualizada do processamento visuoespacial e seus eventos relacionados, à luz de novas técnicas e evidências, com foco em conceitos básicos da organização das funções corticais superiores, suas principais vias e sistemas envolvidos.

**Métodos:**

O estudo foi conduzido em bases de dados nacionais e internacionais LILACS, MEDLINE, SciELO e PubMed; utilizando a palavra "visuoespacial" em combinação com "via", "processamento", "função", "fMRI" e "atenção".

**Resultados:**

Um total de 77 referências consideradas relevantes por sua importância histórica, conceitual e atual foram selecionadas à partir de 1222, incluindo as línguas inglesa, espanhola e portuguesa.

**Conclusão:**

Uma nova construção e funcionamento do processamento visual estão sendo criados, culminando em um modelo baseado em interações dinâmicas e integradas entre vias e sistemas.

## INTRODUCTION

For an individual to thrive and function successfully in the environment, a capacity to interact with the surroundings and react to them is needed in many forms. Among other sensory modalities, such as touch and smell, vision allows us to perform these interactions in a manner that has no match in terms of importance - for sight is a matter of building a self in the world. In this case, numerous papers would be necessary to compile all that is necessary to study the paths and mechanisms involved. However, an overall view on the structures that participate in vision will be addressed here, focusing from the primary visual cortex on. Aiming at that purpose, brief historical references will illustrate how the brain's processes were seen throughout time, embedding a framework for more challenging concepts in vision. Moreover, hierarchical aspects of visual processing are addressed and the general understanding of the visual pathways will be given, focusing on the dorsal and ventral streams, as well as the attentional features that participate in vision.

The goal of this paper is, therefore, to provide a structured perspective on the current organization of sight processing and its anatomical landmarks; harbored on notions of the brain as a composition of systems.

## METHODS

The research was conducted on the basis of national and international electronic databases; LILACS, MEDLINE, SciELO and PubMed. The word "visuospatial" was searched in combination with "pathway", "processing", "function", "fMRI" and "attention". No constriction to date was made and animal studies were included, given the important historical and experimental aspects of this subject.

After abstract reading, a total of 77 references were selected out of 1222; including English, Spanish and Portuguese languages. Reviews were also analyzed and those with updated information or historical impact were included. Criteria for selection considered sources that presented the finding of innovative data prompted at that time, as well as original work of significant historical value and papers dedicated to summarize the bulk of related facts in a comprehensive fashion.

**Hierarchic processing of vision: a challenged perspective?** One could speculate, before getting into the fine details of visuospatial processing, that sight is a continuum of a single process and, thus, should be represented in the same cortical region. That assumption changed as the concept of basic hierarchical processes happening in the brain was developed in time and became assimilated. Such mode of operation became a more comprehensive phenomenon by the time the cerebral cortex was divided into 5 main areas, each of them containing a form of connectivity and cytoarchitectural organization, divided as follows: primary sensory and motor, unimodal associative (modality selective), heteromodal associative, paralimbic and limbic areas.^1^ The last three (heteromodal, paralimbic and limbic), also known as transmodal cortices, were coined together under this term because they are not separated according to a sensory modality.^2^

Furthermore, connections between transmodal areas are reciprocal and abundant, but no same pattern can be found between one unimodal area for a certain sensory modality and the other. This fact reveals a separation on the processing of basic information, maintaining external stimulus free from distortions before it can be refined by heteromodal areas and projected to paralimbic and limbic territories. This influx of information through these areas, also known as bottom-up influence,^3^ exerts influence from the external environment in the internal ambience, culminating in endocrine and autonomic changes. In a way, it is a segregation of the basic procedures going on in the internal and external world previous to their encounter.

Prior to the introduction of the aforementioned model, the assumption that a single region holds the responsibility of coding a certain stimulus was studied in detail throughout history. In the 1870s, the notion of hierarchical and localized brain functions was definitively presented by John Hughlings Jackson, the founder of modern British neurology. Empirically speaking, this question was the object of many lesion studies before and after his time, in a period when clinical observation was the main tool at hand. Through famous cases such as that of Paul Broca's stroke patient,^4^ Phineas Gage,^5^ H.M^6^ and so many others; physicians could reach for a grasp on how localized a brain activity could be.

Also involved in the progress of how connections were conceived are notorious names of neurosciences such as Golgi, Ramon y Cajal and Lorente de Nó.^7^ Animal studies and post-mortem correlations consisted of the principal methods used then, allowing these devoted individuals to start composing the brain as an assembly of systems that holds different specializations. This was a revolutionary statement at a certain point in time, because higher mental processes had once been said, by prominent scientists of that day, to occur indistinctively at the whole of the brain structure. Such thought was still vivid during the first half of the 20^th^ century, despite knowledge pointing to the opposite direction.^8^ It took the seminal work by Norman Geschwind in 1965, later called neo-associationism, to reinstate the notion of the brain as a composition of specialized systems.^9,10^

On the cellular level, the most accepted concept of functioning came to be the so called columnar organization of neurons.^11^ The basic six layered cortex has the same cytoarchitectonic pattern in most cortical areas, turning into a double layer arrangement as it enters paralimbic and limbic areas, more so rostrally.^12^ Despite these topographical nuances, controversies and critical reviews on this matter,^13^ an operation mode was identified in these neuronal gatherings that served to the same specialized area, not necessarily in the same region. For instance, when a stimulus was provided to a primary sensory area, a group of neighboring cells fired together and acted as a unified module. Even more, adjacent modules could present the same firing pattern.^14^ Therefore, in a neuronal level; the networks harbored in different specialized areas are clustered neurons, grouped in modules of cells, working as functional units of a whole.

Following the same evolution and tendencies described above, sight was being described at first as a strict and hierarchically sequenced event, beginning at the striate cortex (V1).^15^ It was also depicted as necessarily passing through different levels of complexity in an orderly fashion,^16^ obeying a step-by-step distribution from primary to unimodal and multimodal cortical areas. In the years to come, the visual cortex of the human brain was described as having a retinotopic organization, based on visual maps originated from the visual fields.^17^ Starting at the retina; this topographic organization was said to be designed from inputs that reached V1, where neighboring receptive fields (RFs) represented adjacent regions of the visual field. As hierarchical areas would grow in complexity from V1 on, subsets of the associative visual cortex were found (i.e. V2, V3, V4).^18^

The first organizational cast that was established based on these findings, would not allow information to reach a higher cortical (V4) level before submitting itself to an intermediate one (V2). Thus, information responsible for basic shapes (e.g. horizontal lines) that was encoded in V1, could not be directly sent to V^4^. The proposition hypothesized at that period affirmed that information had to ascend from small RFs into larger ones,^19,20^ providing the idea that RFs in higher cortical orders were but an intertwinement of basic information encoded in its simplest presentation from lower orders.^21^ Grounded on these principles, theories of two separate streams, the so called dorsal (DP) and ventral pathways (VP), were implicated on visuospatial perception from animal studies as circuits that worked in parallel but not finely integrated.^22^

Presented at first as systems with distinct localizations, relations, characteristics and purposes; the DP was then related to spatial localization (where is it?) and the VP associated to the identification of a stimulus (what is it? what is the name? purpose?).^23^ The dorsal pathway was said to travel from V1 to the parietal cortex, with a further extension to the dorsolateral prefrontal cortex; and the ventral pathway a connection between the occipital and temporal lobes.^24^ These conventions were designed with proper evidence at that time, but recent developments regarding them are changing the classical "two streams-two separate systems" paradigm.

With time and technological advances, a critical and novel analysis was built from the neuroanatomical knowledge accumulated so far, through a behavioural prism. This perspective on specialized areas, interconnected as networks, each of which sheltering its own singular purpose, created the basis for the model accepted today. This overwhelming amount of knowledge has been skyrocketed by modern non-invasive studies by means of functional magnetic resonance imaging (fMRI), diffusion tensor imaging (DTI), diffusion spectrum imaging (DSI), transcranial magnetic stimulation and other advanced scientific arsenal.^25^

Nowadays, a stage wise, but not strictly hierarchical flow of information, with a reciprocal arrangement, seems to be a better explanatory model.

**The dorsal and ventral streams updated: a dynamic and integrated paradigm.** It is known now that there is a great deal of connectivity and converging points shared by the DP and VP. Even in early visual areas belonging to each of these systems, interference through feedback connections can happen reciprocally.^26^ Not only that, it appears that beyond early visual areas, the dorsal and ventral streams directly connect to each other via lateral intraparietal and inferior temporal areas,^27^ as well as the medial temporal region. A point of mutual projections would be the medial temporal lobe (MTL); composed by the hippocampus, rhinal and parahippocampal cortices; where spatial navigation and learning develop.^28,29^ This intertwinement between DP and VP is a proof of how integrated these two systems actually are, demanding a more and more updated integrative paradigm.

On a recent review by Kravitz et al.,^30^ the occipito-parietal network found in the DP is explained to promote a bridge for the distribution of visual information towards higher areas of processing, which in turn are responsible for navigation, visually-guided action, somato-sensation, spatial audition and spatial working memory.^31^ The practical significance of these modalities is an attempt to create spatiotemporal relationships and establish the associations between the items that compose a dynamic scenario, generating grounds for a guided reward-based behavior.^32^ Moreover, with time, long-term representations of how to deal with the environment are found within the posterior parietal cortex (e.g. praxis and tool usage). Also, a continuing process of incorporating a number of non-visual functions takes place within the DP (e.g. number, sequences, melody, and prosody). In this sense, the DP is gaining a "how", rather than just "where", aspect of relating to the external environment.

Both spatial perception and visually guided actions are equally represented in the dorsal stream, developing relations to several areas of the cortex such as frontal, temporal and limbic lobes. There are a few anatomical circuits that can be identified in the dorsal stream. The first one is the occipito-parietal circuit,^33^ which represents projections from early visual areas to the posterior parietal cortex (PPC), including medial aspects of the superior parietal lobule (SPL). This is considered a common anatomical precedent of three other pathways, identified as the parieto-frontal, parieto-premotor and parieto-medial temporal pathways; respectively responsible for spatial working memory, visually guided action and spatial navigation.

The parieto-frontal pathway links the occipito-parietal circuit with areas of the pre-frontal cortex related to top-down modulation of eye movements and spatial working memory.^34^ The second pathway, the parieto-premotor, has two parallel branches that targets the dorsal pre-motor cortex and the ventral pre-motor cortex,^35,36^ resulting in mediation of eye movements, reaching and grasping; not to mention numerous other visually guided actions.

Lastly, the parieto-medial temporal pathway is considered the most complex of them,^37^ presenting direct and indirect projections of information flowing from the inferior parietal lobule (IPL)^38^ to the medial temporal lobe (MTL), the latter including the hippocampus. Also involved in this elaborate complex are the posterior cingulate cortex (PCC), participating in eye movements, attention and navigation;^39-41^ as well as the retrosplenial cortex (RSC),^42^ which has a role in spatial memory, imagination and planning.

All this circuitry located in the parietal-medial temporal path has as a final target the hippocampal formation. This relation permits the complex spatial processing for navigating in the environment, casting relevant information on distant-space perception and various frames of reference (whole-body motion, head direction and spatial long-term memory).^43,45^ The DP, therefore, assembles spatial information and directs behavior through vision.

Kravitz^30^ explains the ventral pathway as an occipto-temporal road from V1 to cortical and subcortical structures, implicated in learning and memory by means of visual information. The paramount characteristic found here lies on specific representations glued together over associations of stable aspects of visual data. Features such as shape, color, size, and brightness; available in the striate cortex, are the most basic informations used. These dimensions might be readily available in the input (e.g. retinotopic position, brightness) or they may be a conjunction of basic dimensions (e.g. shape, faceness). The VP has long been responsible for assigning meaning to visual information.^46^ Coursing through various routes from early visual areas to the anterior inferior temporal cortex (aIT), a modern conception of this circuitry is being described as a region for different forms of object quality processing along a recurrent and highly interactive network.

The previous paradigm built for the VP was of a computational model of processing, which encompassed a strict hierarchical sequence of steps that restricted information at specific levels. In this chain of events, information could not flow from a most basic area (V1) to a high order one (V4) before being processed by an intermediate (V2). It is believed now that a reciprocal interaction in the shape of feed-forward and feed-back projections is a more plausible manner of functioning, some of them actually bypassing intermediate areas and going straight to late stages of the hierarchy.^47^ Other recent evidence points out that the retinotopic organization can be found even in high levels of object representations^48^ and, in addition, that a vast network links at least six different cortical and subcortical structures to the ventral stream.

These wirings are separated as follows:^49^ occipito-temporo-neostriatal pathway, involved in stimulus-response associations; occipito-temporo-amygdaloid pathway, related to emotionally salient stimuli; occipito-temporo-ventral striatum pathway, conferring valence to stimulus; occipito-temporo-medial temporal, occipito-temporo-orbitofrontal, and occipito-temporo-ventro-lateral prefrontal pathways. These last three are aligned, respectively, with long-term memory, object-reward association, and object working memory.

Furthermore, the VP is a path that travels in a previously known "central route", going from V1 to V2 and then to the inferior temporal cortex, coursing in an anterior direction towards the temporal pole. However it also displays an alternative route that passes through the superior temporal sulcus (STS) in its fundus and ventral bank.^50^ This configuration makes possible for information to travel by alternative ways, reaching the most anterior aspect of the inferior temporal lobe. By these routes, key aspects of the VP, in its utmost property, result in highly complex visual representations for several crucial aspects of human functionality and behavior, such as the face area.^51^

In a way, even though the classical attributions of each pathway remain largely preserved, a new concept deriving from recent knowledge points towards the notion that spatial dimensions can contribute to ventral pathway representations and some aspects of object shape are necessary in the dorsal pathway to effectively guide action.^52,53^

Although the DP and VP are, therefore, progressively associated in a mutual effort for perceiving an object in space and qualifying it, the amount of importance an individual attributes to such information falls on attentional processes.

**Attention as a modulatory system of visual processing.** The bulk of information a visual stimulus bears is enormous. Humans put together these data by successive small eye fixations on the environment, promoted by saccadic movements,^54^ that are controlled both by cortical^55-57^ and subcortical structures.^58^ The myriad of details that are extracted from the external world needs to be screened in a manner that would allow an individual to select only relevant facts and perform a guided behavior. In that sense, the conjunction of filtering information for a given purpose and the limited capacity of processing that can be handled by the brain, requires a finely tuned mechanism.^59^ Attention is the tool in which this responsibility falls, consisting of an anatomically separated entity from processing systems^60^ and holding influence from top-down and bottom-up interactions.^61^

Top-down influences are based on the observers "internal" experience, much concerned with one's intention, constituted by past experiences and expectations over that context and scenario. Bottom-up influences, on the other hand, are based on facts that are external to the observer, mainly built from stimulus salience^62^ which, by its turn, represents the degree of attracting one's attention based on basic features from the visual map. In another way to put it, top-down (endogenous attentional control) is what you expect and bottom-up (exogenous attentional control) is the summation of basic physical characteristics of items in a visual display.^63^ Consequently, visual attention can be voluntarily directed to goal-driven purposes, such as looking for something you lost at a public place, however remaining open to random salient stimuli, like a flash of light.^64^

Attention is segmented in three major pillars:^65^ alerting, a strongly lateralized resource that is responsible for general sustained vigilance; orienting and executive attention. The last two work synergistically in real-world sets, gathering information on both when and where a target will occur.^66^ Orienting participates in the capability to prioritize sensory input by selecting a modality or location. It is placed at frontal and posterior areas, respectively the frontal eye fields (FEF),^67,68^ where saccades are also influenced;^69^ and the posterior parietal cortex (intraparietal sulcus).^70^ Still on orienting attention, it seems that the temporo-parietal junction (TPJ),^71^ especially on the right side and along with the ventral frontal cortex, is heavily implicated on breaking attention to a currently attended location so that reorienting can take place.^72^ It is presumed that dorsal areas of the parietal cortex, including the SPL, are involved in top down attentional orienting, while ventral regions including the TPJ are involved in bottom-up attentional orienting.^73^ This fronto-parietal system is concerned, then, with start-cue aspects of vision and is supposed to relate with task initiation and switching.

After orienting takes place, capturing awareness of a certain target is relied on executive attention, promoting impoverishment in the detection of another unimportant one.^74^ The executive attention is anatomically referenced to the anterior cingulate cortex,^75^ midline cortex and the insula.^76^ It seems to act as a stable background maintainer for task performance as a whole, managing conflicts tasks and emotional aspects of the ongoing process.^77^

For the matter of visual attention, the DP seems to be more prominent than the VP and both visual and attentional systems have to work together in an effort to permit intake of visual stimulus in an organized and selected way.

### Conclusion.

Lastly, one could say that the variables included in vision and its related processes are numerous. Being able to study the synergism of so many areas, gathered under a model of integrative and dynamic relations within visual processing is a learning to be built constantly. Aside the main pathways discussed throughout this paper, other structures and mechanisms are also involved in vision, but would require more than could be summarized within this article.

Nevertheless, the main goal to be achieved within this review is a general understanding of the pathways and systems participating in vision, creating notions for more specific knowledge on how cognitive tests, neurological syndromes and pathologies are found within these structures.
